# Structure-changeable luminescent Eu(III) complex as a human cancer grade probing system for brain tumor diagnosis

**DOI:** 10.1038/s41598-023-50138-9

**Published:** 2024-01-22

**Authors:** Mengfei Wang, Masaya Kono, Yusaku Yamaguchi, Jahidul Islam, Sunao Shoji, Yuichi Kitagawa, Koji Fushimi, Sora Watanabe, Go Matsuba, Akihisa Yamamoto, Motomu Tanaka, Masumi Tsuda, Shinya Tanaka, Yasuchika Hasegawa

**Affiliations:** 1 Institute for Chemical Reaction Design and Discovery (WPI-ICReDD), Hokkaido University, Sapporo, Hokkaido 001-0021 Japan; 2 Graduate School of Chemical Sciences and Engineering, Hokkaido University, Sapporo, Hokkaido 060-8628 Japan; 3 Faculty of Engineering, Hokkaido University, Sapporo, Hokkaido 060-8628 Japan; 4 Department of Engineering, Nara Women’s University, Nara, 630-8506 Japan; 5 Graduate School of Organic Material Engineering, Yamagata University, Yonezawa, Yamagata 992-8510 Japan; 6 Center for Integrative Medicine and Physics, Institute for Advanced Study, Kyoto University, Kyoto, 606-8501 Japan; 7 Physical Chemistry of Biosystems, Institute of Physical Chemistry, Heidelberg University, 69120 Heidelberg, Germany; 8 Department of Cancer Pathology, Faculty of Medicine, Hokkaido University, Sapporo, Hokkaido 060-8638 Japan

**Keywords:** Diagnostic markers, Fluorescent probes

## Abstract

Accurate determination of human tumor malignancy is important for choosing efficient and safe therapies. Bioimaging technologies based on luminescent molecules are widely used to localize and distinguish active tumor cells. Here, we report a human cancer grade probing system (GPS) using a water-soluble and structure-changeable Eu(III) complex for the continuous detection of early human brain tumors of different malignancy grades. Time-dependent emission spectra of the Eu(III) complexes in various types of tumor cells were recorded. The radiative rate constants (*k*_r_), which depend on the geometry of the Eu(III) complex, were calculated from the emission spectra. The tendency of the *k*_r_ values to vary depended on the tumor cells at different malignancy grades. Between T = 0 and T = 3 h of invasion, the *k*_r_ values exhibited an increase of 4% in NHA/TS (benign grade II gliomas), 7% in NHA/TSR (malignant grade III gliomas), and 27% in NHA/TSRA (malignant grade IV gliomas). Tumor cells with high-grade malignancy exhibited a rapid upward trend in *k*_r_ values. The cancer GPS employs Eu(III) emissions to provide a new diagnostic method for determining human brain tumor malignancy.

## Introduction

Cancer is a major public health problem in every country of the world^1,2^. Increasing the universal awareness of early cancer diagnosis is key to increasing the chances of successful treatment^3–5^. Bioimaging technologies based on luminescent molecules are powerful approaches for locating and distinguishing tumor cells. Luminescent molecules have been developed as non-invasive probes for early cancer diagnosis. Luminescent organic dyes exhibit tunable fluorescence properties associated with structural modifications. Pu and Yuan summarized recent studies on near-infrared (NIR) shifting fluorescence using structurally modified hemicyanine dyes for the bioimaging and diagnosis of cancers in mice^6,7^. Urano reported a membrane-permeable hydroxymethyl rhodol derivative for fluorescence-guided diagnosis of ovarian cancer in mice^8,9^. Metal-free thermally activated delayed fluorescence (TADF) materials are attractive next-generation organic dyes for biomedical applications. Hudson and Algar described red-emissive TADF polymer dots for time-gated cellular imaging of human liver cancer cells^10,11^. Among luminescent molecules, transition metal complexes show potential advantages in bioimaging and cancer diagnosis owing to their long phosphorescence lifetimes. Ma and Leung developed design strategies for transition-metal-complex-based cancer diagnosis^12,13^. Thomas et al. mainly concentrated on phosphorescent Ru(II) complexes that bind DNA and other biomolecules such as cell probes, therapeutics, and theranostics^14^. Luminescent lanthanide complexes with long-lived 4f–4f transitions have also been reported for biomedical diagnoses^15^. Parker and Bünzli reviewed the current developments in water-soluble lanthanide(III) cyclen- and triazacyclononane-based complexes for cell imaging and bioanalysis and in biosensors, pH detectors, and selective-ion probes^15–20^.

Here, we focused on a structure-changeable Eu(III) complex for early tumor diagnosis. We previously demonstrated that the radiative rate (*k*_r_) constants of Eu(III) complexes are dependent on their geometrical coordination structures^21,22^. Flexible structural changes in the Eu(III) complex are promoted in tumor cells, resulting in changes in their *k*_r_ constants. The changeable *k*_r_ constant is expected to enable continuous detection of tumor activity and growth processes.

**Figure 1:**
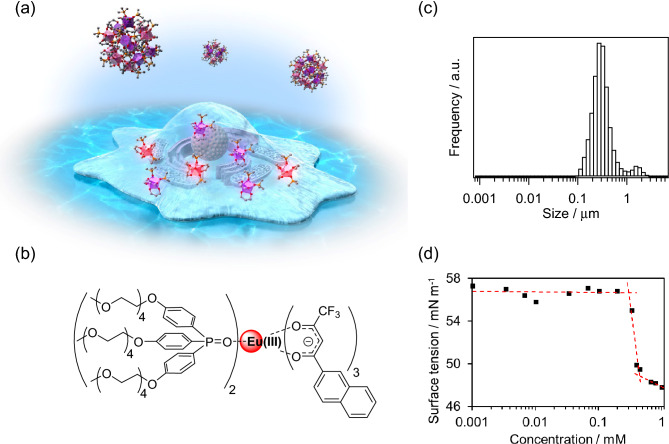
(**a**) Concept of this work: Eu(III) complex was designed for cellular imaging and cancer diagnosis; (**b**) Molecular structure of [Eu(ntfa)_3_(TEGPO)_2_]; (**c**) DLS measurement. Particle size distribution of 1 mM [Eu(ntfa)_3_(TEGPO)_2_] in water/methanol mixture (90/10 v/v); (**d**) CAC determination. Relationship between [Eu(ntfa)_3_(TEGPO)_2_] concentration and its surface tension.

In this paper, we report a human cancer grade probing system (GPS) using water-soluble and structurally changeable Eu(III) complexes (Fig. 1a,b). In this system, the activities of early human brain tumors were evaluated using the *k*_r_ constant of the structure-changeable Eu(III) complex. To mimic the malignant transformation of human glioma of grades II, III, and IV, normal human astrocytes (NHA) were transformed through serial gene transfer of *hTERT* (T), *SV40ER* (S), *H-RasV12* (R), and *myrAKT* (A). The transformed cells were designated NHA/TS, NHA/TSR, and NHA/TSRA cells, representing grade II, III, and IV glioma, respectively^23^. Triphenylphosphine oxide attached to tetraethyleneglycol methylether (TEGPO) was selected as a coordination ligand to improve water solubility and protection from amino acid molecules in the cell culture medium (DMEM: Dulbecco’s Modified Eagle Medium^23,24^). The luminescence properties of the designed Eu(III) complex were studied in methanol, a water/methanol mixture (90/10 v/v), and DMEM. The time-dependent emission spectra of the Eu(III) complex in human brain tumors of different malignancy grades were recorded. The geometry-dependent *k*_r_ constants of the Eu(III) complexes were evaluated using the emission spectral shapes. In this study, we observed that the *k*_r_ constants in cancer cells show different and varying trends for different malignancy grades of the tumors. The cancer GPS, which uses a structure-changeable luminescent Eu(III) complex, provides a new analytical method for early diagnosis of human brain tumors.

## Results and discussion

### Molecular design and structural evaluation

A luminescent Eu(III) complex with π-expanded β-diketonate (ntfa: 3-(2-naphthoyl)-1,1,1-trifluoro-acetonate; ntfa) and bis[2-(diphenylphosphino)phenyl] ether oxide (DPEPO) has been reported^25^. The ntfa and DPEPO ligands offered low vibrational relaxation, excellent thermostability, and high photosensitized energy transfer efficiency for the Eu(III) complex [Eu(ntfa)_3_(DPEPO)_2_]. Furthermore, the Eu(III) complex [Eu(ntfa)_3_(H_2_O)_2_] with the ntfa ligand showed bright red luminescence when observed using bioimaging confocal optical microscope systems (excitation wavelength: 405 nm)^26^. To develop a water-soluble Eu(III) complex for tumor diagnosis, tetraethyleneglycol methylether-attached triphenylphosphine oxide (TEGPO) was introduced into the Eu(III) complex to improve its solubility in aqueous solutions^27^. A new water-soluble Eu(III) complex, [Eu(ntfa)_3_(TEGPO)_2_], containing hydrophobic ntfa and hydrophilic TEGPO ligands was designed for cellular imaging and cancer diagnosis (Fig. 1a,b).

[Eu(ntfa)_3_(TEGPO)_2_] which contains both hydrophobic and hydrophilic groups, is expected to exist in a micelle-like form in aqueous solutions. To determine the state of the spherical micelle-like aggregates, the size distribution of 1 mM [Eu(ntfa)_3_(TEGPO)_2_] in a water/methanol mixture (90/10 v/v) was measured using dynamic light scattering (Fig. 1c). The mean volume diameter of [Eu(ntfa)_3_(TEGPO)_2_] aggregates was estimated to be 0.37 μm at room temperature. Surface tension measurements were performed over a range of [Eu(ntfa)_3_(TEGPO)_2_] concentrations in a water/methanol mixture. The critical aggregate concentration (CAC) of [Eu(ntfa)_3_(TEGPO)_2_] in a water/methanol mixture (c*_MeOH/H2O_ = 0.45 mM), was determined from the intercept of the linear fit of the plot shown in Fig. 1d^28^. Notably, the surface tension remained constant up to the concentration of approximately 0.3 mM. The constant surface tension of approximately 57 mN/m observed here is much lower than that of pure water (73 mN/m) and agrees well with the surface tension of water containing 10 wt% (and hence, 12.5% v/v) methanol^29^. However, a 200 μL portion of 1 mM [Eu(ntfa)_3_(TEGPO)_2_] is injected into 2 ml of the cell culture medium in the experiments with glioblastoma cells. This indicates that the methanol fraction in the [Eu(ntfa)_3_(TEGPO)_2_] suspension was not 10 vol%. To determine the surface activity of [Eu(ntfa)_3_(TEGPO)_2_] in cell experiments, we measured the surface tension of [Eu(ntfa)_3_(TEGPO)_2_] under systematic dilution of a water/methanol mixture in water (Supplementary Fig. 1, red). For comparison, the surface tension of the water/methanol mixture without [Eu(ntfa)_3_(TEGPO)_2_] is plotted (blue). The surface tension in the presence of [Eu(ntfa)_3_(TEGPO)_2_] (red) decreases monotonically, with a kink at 0.36 mM. However, the surface tension of water/methanol mixture with no [Eu(ntfa)_3_(TEGPO)_2_] (blue) remained near 73 mN until 0.13 mM, suggesting that methanol does not affect the surface tension of water up to this concentration. Therefore, we calculated the surface excess concentration (Γ = 615 nmol/m^2^) and surface area per molecule at the interface (A = 270 Å^2^) from the slope of the plot shown in Supplementary Fig. 1^30,31^. The obtained data suggest that the concentration of [Eu(ntfa)_3_(TEGPO)_2_] in the cell experiments is approximately 100 μM, at which [Eu(ntfa)_3_(TEGPO)_2_] molecules are both in the solution and at the interface. Ultrasmall- and small-angle X-ray scattering (USAXS/SAXS) profiles also showed the presence of [Eu(ntfa)_3_(TEGPO)_2_] aggregates with radii of approximately 150 nm (Supplementary Fig. 2). This structural information in the water/methanol mixtureindicates that the particle size of the [Eu(ntfa)_3_(TEGPO)_2_] aggregates was larger than that of the Triton X-100 micelles with Eu(III) complexes (Supplementary Fig. 3). The formation of large particles plays an important role in maintaining the stability of the Eu(III) complex in DMEM^32,33^.

### Luminescence properties

**Figure 2:**
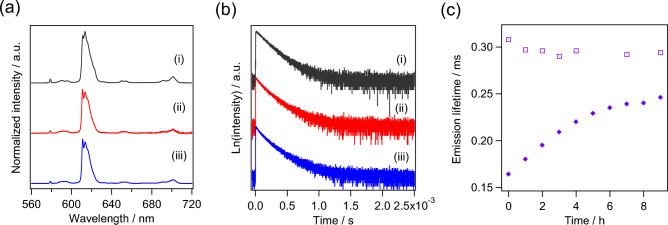
Luminescence properties. (**a**) Emission spectra and (**b**) emission lifetimes of [Eu(ntfa)_3_(TEGPO)_2_] in (i) methanol, (ii) water/methanol mixture (90/10 v/v) and (iii) DMEM mediums. (**c**) Time courses of emission lifetimes of [Eu(ntfa)_3_(TEGPO)_2_] (□) and [Eu(ntfa)_3_(H_2_O)_2_] (♦) in DMEM medium.

The emission spectra of 0.1 mM [Eu(ntfa)_3_(TEGPO)_2_] in methanol, water/methanol mixture (90/10 v/v), and DMEM are shown in Fig. 2a. The emission bands at approximately 578, 592, 613, 650, and 698 nm are assigned to the characteristic ^5^*D*_0_ → ^7^*F*_*J*_ (*J* = 0, 1, 2, 3, and 4) transitions of Eu(III).

The emission lifetime decays of 0.1 mM [Eu(ntfa)_3_(TEGPO)_2_] in methanol, water/methanol mixture (90/10 v/v), and DMEM are also described in Fig. 2b and Table 1. The emission profiles exhibited a double exponential decay with the average emission lifetime in DMEM (0.27 ms) being similar to those in methanol (0.28 ms) and water/methanol mixture (0.27 ms). The intrinsic emission quantum yield Φ_*f–f*_, radiative rate (*k*_r_), and nonradiative rate (*k*_nr_) constants were calculated using the following Eqs. ^34,35^.

$$k_{{\text{r}}} = \frac{1}{{\tau_{{{\text{rad}}}} }} = A_{{\text{MD,0}}} n^{3} \frac{{I_{{{\text{tot}}}} }}{{I_{{{\text{MD}}}} }}$$

$$k_{{{\text{nr}}}} = \frac{1}{{\tau_{{{\text{ave}}}} }} - \frac{1}{{\tau_{{{\text{rad}}}} }}$$

$$\Phi_{{\text{f - f}}} = \frac{{k_{{\text{r}}} }}{{k_{{\text{r}}} + k_{{{\text{nr}}}} }} = \frac{{\tau_{{{\text{ave}}}} }}{{\tau_{{{\text{rad}}}} }}$$

where *A*_MD,0_ is the spontaneous emission probability for the ^5^*D*_0_ → ^7^*F*_1_ transition in vacuo (14.65 s^−1^) and *n* is the refractive index of solution (1.33 for methanol and water/methanol mixture, 1.3376 for DMEM)^36,37^. The (*I*_tot_/*I*_MD_) is the ratio of the total area of Eu(III) emission spectrum to the peak area of the ^5^*D*_0_ → ^7^*F*_1_ transition. In addition, the overall luminescence quantum yield of Eu(III) from ligand photoexcitation via intramolecular energy transfer Φ_*tot*_ was measured in methanol^38^. The photophysical parameters are listed in Table 1. The time courses of the emission lifetimes of 0.1 mM [Eu(ntfa)_3_(TEGPO)_2_] and [Eu(ntfa)_3_(H_2_O)_2_] (the precursor of [Eu(ntfa)_3_(TEGPO)_2_]) in DMEM are presented in Fig. 2c. The emission lifetime of [Eu(ntfa)_3_(TEGPO)_2_] was constant, indicating that [Eu(ntfa)_3_(TEGPO)_2_] remained stable in DMEM. In contrast, the emission lifetimes of [Eu(ntfa)_3_(H_2_O)_2_] gradually increased with time, suggesting that the complex was labile and easily underwent the ligand exchange reaction in DMEM. Long-chain TEGPO ligands protect the Eu(III) complex from coordination molecules existing in the DMEM and suppress the ligand-exchange reaction. The use of stable [Eu(ntfa)_3_(TEGPO)_2_] in cancer GPS makes it remarkably simpler and easier to understand the influence of tumor malignancy on Eu(III) luminescence and emission spectra.

**Table 1:** Luminescence properties of [Eu(ntfa)_3_(TEGPO)_2_] in various solutions.

Concentration	Solution	τ_ave_/ms	*k*_r_/s^−1^	*k*_nr_/s^−1^	Φ_*f-f*_/%	Φ_*tot*_/%
0.1 mM	Methanol	0.28	8.1 × 10^2^	2.8 × 10^3^	22	14
	Water/methanol mixture (90/10 v/v)	0.27	6.8 × 10^2^	3.0 × 10^3^	19	–
	DMEM	0.27	7.1 × 10^2^	2.9 × 10^3^	20	–
1 mM	Methanol	0.29	7.2 × 10^2^	2.7 × 10^3^	21	15
	Water/methanol mixture (90/10 v/v)	0.29	7.3 × 10^2^	2.8 × 10^3^	21	–

### Early tumor diagnostic applications

**Figure 3:**
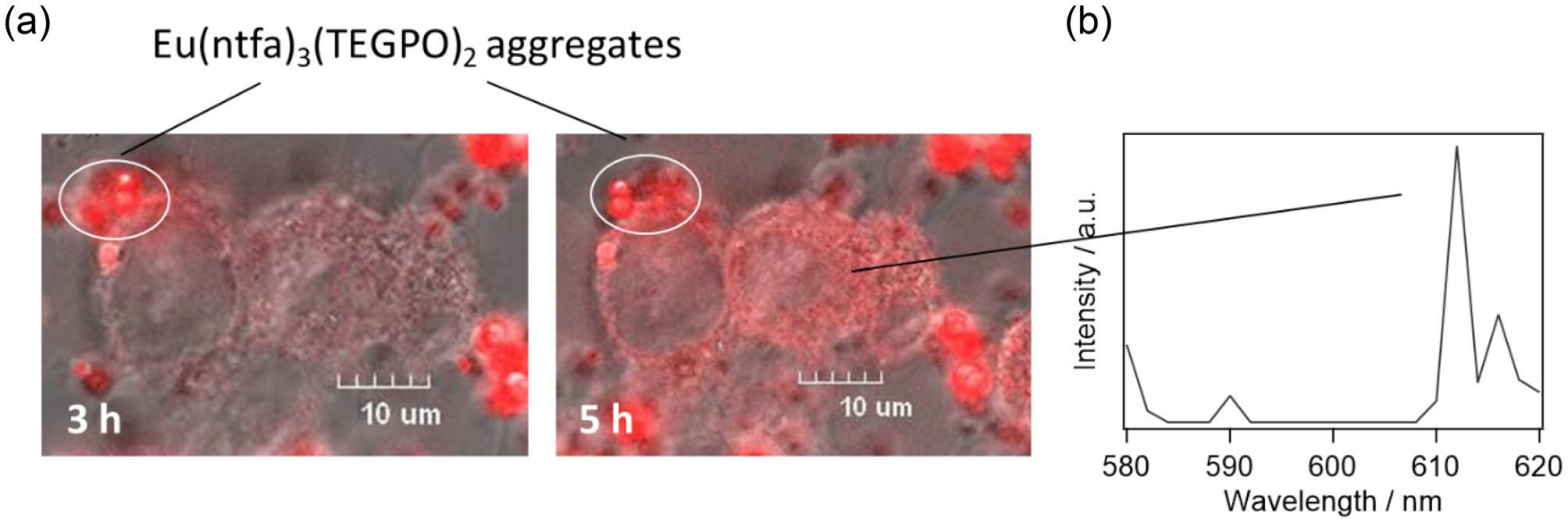
(**a**) Zoomed-in confocal images and (**b**) λ-scan image of NHA/TSRA cells treated with [Eu(ntfa)_3_(TEGPO)_2_].

Confocal images of NHA-based glioma model cells (NHA/TS, NHA/TSR, and NHA/TSRA) after the injection of [Eu(ntfa)_3_(TEGPO)_2_] into DMEM are shown in Fig. 3 and Supplementary Fig. 4. Microspheres were observed on the tumor cell membranes (Fig. 3a)^39^. The luminescence intensity of the tumor cells after 5 h was brighter than that after 3 h. The red spheres and luminescent cells were derived from [Eu(ntfa)_3_(TEGPO)_2_] aggregates in DMEM and molecules in NHA/TSRA, respectively. To determine the origin of the intracellular luminescence, the λ-scan images of NHA/TSRA cells were acquired using a confocal laser scanning microscope (Fig. 3b). The characteristic 4f–4f emission band associated with the ^5^*D*_0_ → ^7^*F*_2_ transition was observed at approximately 610 nm, indicating that [Eu(ntfa)_3_(TEGPO)_2_] entered and stained the tumor cells. We hypothesized that cellular [Eu(ntfa)_3_(TEGPO)_2_] uptake was induced by endocytosis in tumor cells (Supplementary Fig. 5)^40–42^.

**Figure 4:**
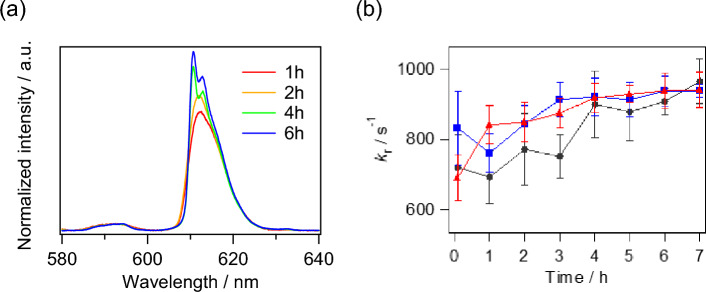
(**a**) Time-dependent emission spectra of [Eu(ntfa)_3_(TEGPO)_2_], pertaining to its uptake in NHA/TSR cells; (**b**) time-course of *k*_r_ values in NHA/TS (●), NHA/TSR (■), and NHA/TSRA (▲) cells.

The emission spectra of [Eu(ntfa)_3_(TEGPO)_2_] in NHA/TS (benign grade II glioma), NHA/TSR (malignant grade III glioma), and NHA/TSRA (malignant grade IV glioma) cells are shown in Fig. 4a and Supplementary Fig. 6. The intensity of the emission band associated with the ^5^*D*_0_ → ^7^*F*_2_ transition (610 nm) increased with increasing tumor cell invasion time. The radiative rate constants (*k*_r_) of the Eu(III) complexes in the tumor cells were calculated from the cellular emission spectra. The time courses of *k*_r_ values (mean ± SD) in NHA/TS (benign grade II gliomas), NHA/TSR (malignant grade III gliomas), and NHA/TSRA (malignant grade IV gliomas) cells are shown in Fig. 4b. At T = 0 h, the *k*_r_ of [Eu(ntfa)_3_(TEGPO)_2_] displays similar values in different tumor cells (720 ± 94 s^–1^ in NHA/TS, 833 ± 106 s^–1^ in NHA/TSR, and 690 ± 65 s^–1^ in NHA/TSRA). Different trends in the *k*_r_ values were observed with increasing invasion time for different tumor cells. At T = 1 h, the *k*_r_ values were estimated to be 693 ± 75 s^–1^ in NHA/TS (benign grade II gliomas), 731 ± 75 s^–1^ in NHA/TSR (malignant grade III gliomas), and 842 ± 54 s^–1^ in NHA/TSRA (malignant grade IV gliomas). Furthermore, during the time interval between T = 0 and T = 3 h, the *k*_r_ values exhibited a respective increase of 4%, 7%, and 27% in NHA/TS, NHA/TSR and NHA/TSRA. These data indicate that the change in *k*_r_ values depends on the malignancy grade of the tumors. After T = 4 h, the *k*_r_ values became nearly consistent regardless of the tumor type, suggesting self-assembly of Eu(III) complex within cells or cell death. The occurrence of cell death with Eu(III) complex treatment was verified through cell counting measurements (Supplementary Fig. 8). Generally, the *k*_r_ value is affected by the coordination geometry of the trivalent lanthanide complexes^21,22,43^. In DMEM, the [Eu(ntfa)_3_(TEGPO)_2_] aggregates showed no structural changes and the *k*_r_ values remained constant (Supplementary Fig. 7). From these aspects, we infer that there are structural changes in the [Eu(ntfa)_3_(TEGPO)_2_] single molecules after their uptake into the tumor cells (Fig. 1a). The interaction with [Eu(ntfa)_3_(TEGPO)_2_] appears to be influenced by the degree of malignancy, as reflected in the alterations to the coordination geometry of single Eu(III) molecules. High-grade malignant tumors promote flexible structural changes in the Eu(III) complex and increase the *k*_r_ values. Inversely, Eu(III) complex affected tumor cell cycle distribution and lead to increases in apoptotic cells after T = 3 h (Supplementary Fig. 9), providing possible explanation for cell death occurrence.

Diagnosing cancer, especially in the early stages of the disease, is difficult^44,45^. For example, biomarker testing is a feasible method for discovering cancer^46–49^, and in most cases, cancer biomarkers appear at detectable levels until advanced stages of the disease^50^. Circulating tumor cell observation using luminescent Eu(III) nanoparticle-based nanoprobes enables early cancer diagnosis without malignancy determination^51,52^. According to the experimental results, the structural conformations and emission properties of [Eu(ntfa)_3_(TEGPO)_2_] readily respond to human brain tumor malignancies. The developed Eu(III) complex is capable of rapid and precise determination of human tumor malignancy quickly and exactly.

## Conclusion

In this study, we demonstrated a human cancer grade probing system (GPS) using a new water-soluble and structure-changeable Eu(III) complex for early brain tumor diagnosis. The designed Eu(III) complex containing π-expanded β-diketonates and tetraethyleneglycol methylether-attached triphenylphosphine oxides was present in micelle-like aggregates and remained stable in the DMEM cell culture medium. In cell experiments, the *k*_r_ values exhibited an increase of 4% in NHA/TS (benign grade II gliomas), 7% in NHA/TSR (malignant grade III gliomas), and 27% in NHA/TSRA (malignant grade IV gliomas) during invasion time between T = 0 and T = 3 h. The increasing trend of the *k*_r_ values of the Eu(III) complex in tumor cells depends on the tumor malignancy grade, suggesting that different tumor activities and their growth processes may be the origin of the geometrical coordination changes of the Eu(III) complex. High cellular activity, that is, rapid cellular uptake of the Eu(III) complex in DMEM, facilitates the transformation from aggregates to single molecules by altering the Eu(III) coordination conformation. In addition, substances in the cell body can induce conformational changes in the Eu(III) complexes. This cancer GPS, which uses a structure-changeable luminescent Eu(III) complex, provides a new diagnostic method for determining human brain tumor malignancy.

## Methods

### Materials

All chemicals were of reagent grade and used without further purification. Fourier transform infrared (FT-IR) spectroscopy was performed using a JASCO FT/IR-4600 spectrometer. ESI–MS measurement was performed using a JEOL JMS-T100LP instrument. NMR spectra were recorded on a JEOL ESZ-400S FT spectrometer, operating at 400 MHz (1H), 376 MHz (19F), and 162 MHz (31P). Reference for ^31^P NMR chemical shifts was H_3_PO_4_. DLS measurements were performed with a Microtrac Nanotrac Wave II-UT151. CMC was determined by Wilhelmy plate method using KRŰSS K20 EasyDyne. The confocal images were obtained using confocal laser scanning microscopy, Olympus FV3000-IX83 (excitation: 405 nm, emission: 580–620 nm).

### Synthesis of [Eu(ntfa)_3_(TEGPO)_2_]

[Eu(ntfa)_3_(H_2_O)_2_] (0.12 g, 0.12 mmol) and TEGPO (0.43 g 0.47 mmol) were added in dichloromethane (12 ml). The reaction solution was stirred at room temperature for 6 h. The reaction solution was filtered (0.45 μm) to remove unreacted [Eu(ntfa)_3_(H_2_O)_2_] and concentrated under reduced pressure. The crude product was washed by *n*-hexane (100 mL) and hot *n*-hexane (50 °C). Small amount methanol was added. The resulting solution was filtered (0.45 μm) and concentrated under reduced pressure to give a light-yellow oil as the desired product (0.17 g, 0.06 mmol, yield 49%).

^19^F NMR(376 MHz, CDCl_3_): d/ppm = − 80.80 (s).

ESI-Mass (m/z): [M + Na]^+^calcd. for C_132_H_162_EuF_9_NaO_38_P_2_, 2763.92; found, 2764.01.

FT-IR (ATR): 1617 (st, C=O), 1117 (st, P=O) cm^−1^.

### Ultra-small and small angle X-ray scattering (USAXS/SAXS)

The USAXS/SAXS profiles of [Eu(ntfa)_3_(TEGPO)_2_] in methanol, water/methanol mixture (90/10 v/v) and DMEM mediums were measured using the BL19B2 beamline at SPring-8 at an incident X-ray beam wavelength of 0.068 nm. The camera lengths for USAXS and for SAXS were set to 40.77 m and 3.04 m, respectively. The 2D USAXS/SAXS profiles were obtained using a PILATUS-2 M two-dimensional detector (Dectris Ltd., Baden, Switzerland). The scattering vector, *q*, was recorded between 4 × 10^–3^ and 3.0 nm^−1^. The solution sample was placed in a 1.0-mm-thick sample cell sandwiched between polyether-ether-ketone films.

### Optical measurements

Emission and excitation spectra of [Eu(ntfa)_3_(TEGPO)_2_] in methanol, water/methanol mixture (90/10 v/v) and DMEM were measured using a Horiba Fluorolog-3 spectrofluorometer and corrected for the response of the detector system. Emission decay profiles were measured using the third harmonics (355 nm) of a Q-switched Nd:YAG laser (Spectra Physics, INDI-50, FWHM = 5 ns, λ = 1064 nm) and a photomultiplier (Hamamatsu Photonics, R5108, response time ≤ 1.1 ns). The Nd:YAG laser response was monitored with a digital oscilloscope (Sony Tektronix, TDS3052, 500 MHz) synchronized to the single-pulse excitation. Emission lifetimes were determined from the slope of logarithmic plots of the decay profiles.

### Cell culture

Normal human astrocyte (NHA) were purchased (Cambrex Bio Science, Walkersville, MD, USA), and NHA-based glioma model cells (NHA/TS, NHA/TSR, NHA/TSRA) were established by introducing with hTERT (T), and SV40ER (S), H-RasV12 (R), and mycAKT (A) as previously reported^53,54^.

NHA-based glioma model cells (NHA/TS, NHA/TSR, NHA/TSRA) were cultured in Dulbecco’s Modified Eagle Medium (DMEM, Nissui Pharmaceutical Co. Ltd., Tokyo, Japan) supplemented with 10% fetal bovine serum (FBS) (Corning, NY, USA), 2 mM glutamine, 100 units/ml penicillin and 100 μg/ml streptomycin (Sigma-Aldrich). Cells were cultured at 37 °C under a humidified atmosphere containing 5% CO_2_.

### Confocal imaging

NHA cells (2 × 10^5^) were plated on a 35 mm glass based dish (IWAKI, 3911-035, Tokyo, Japan) and incubated in DMEM for overnight. The culture medium was replaced with 2 ml phenol red-free DMEM (Gibco, Thermo Fisher Scientific K.K., Tokyo, Japan). 200 μl of 1 mM [Eu(ntfa)_3_(TEGPO)_2_] in water/methanol mixture (90/10 v/v) was dropped into glass based dish in which cells were being cultured. The cells were cultured at 37 °C under a humidified atmosphere containing 5% CO_2_ for bioimaging measurements.

### Measurement of cellular emission spectra

NHA cells (2 × 10^5^) were plated on eight 35 mm culture dishes and incubated in DMEM for overnight. The medium was replaced with 2 ml phenol red-free DMEM. 200 μl of 1 mM [Eu(ntfa)_3_(TEGPO)_2_] in water/methanol mixture (90/10 v/v) was dropped into the six culture dishes. The cells were cultured at 37 °C under a humidified atmosphere containing 5% CO_2_. After addition of [Eu(ntfa)_3_(TEGPO)_2_] in certain time (5-min, 1, 2, 3, 4, 5, 6, 7-h), the NHA cells were washed twice with 2.5 ml PBS, and 200 μl of EDTA-Trypsin was added. The detached cells were collected in additional 300 μl of phenol red-free DMEM. Cell suspensions were transferred into 10 mm optical cell for emission spectra measurements.

### Guideline statement

The authors confirm that all procedures of the experiments complied with relevant institutional, national, and international guidelines and legislation.

### Supplementary Information

Supplementary Information.

## Data Availability

The data that support the findings of this study are available from the corresponding authors on reasonable request.
