# Supplementary material for: Structure-changeable luminescent Eu(III) complex as a human cancer grade probing system for brain tumor diagnosis
Source: Sci Rep. 2024 Jan 22;14:778. doi: 10.1038/s41598-023-50138-9 (PMC10803341; doi:10.1038/s41598-023-50138-9)
Supplement: Supplementary file 1 — Supplementary Information. [file 41598_2023_50138_MOESM1_ESM.docx]

Supporting information for

**Structure-changeable luminescent Eu(III) complex as a human cancer grade probing system for brain tumor diagnosis**

Mengfei Wang,^1,3^* Masaya Kono,^2^ Yusaku Yamaguchi,^2^ Jahidul Islam,^3^ Sunao Shoji,^4^ Yuichi Kitagawa,^1,3^ Koji Fushimi,^3^ Sora Watanabe,^5^ Go Matsuba,^5^ Akihisa Yamamoto,^6^ Motomu Tanaka,^6,7^ Masumi Tsuda,^1,8^ Shinya Tanaka,^1,8^ Yasuchika Hasegawa^1,3^*

^1^*Institute for Chemical Reaction Design and Discovery (WPI-ICReDD), Hokkaido University, Sapporo, Hokkaido, 001-0021, Japan*

^2^*Graduate School of Chemical Sciences and Engineering, Hokkaido University, Sapporo, Hokkaido, 060-8628, Japan*

^3^*Faculty of Engineering, Hokkaido University, Sapporo, Hokkaido, 060-8628, Japan*

*^4^Department of Engineering, Nara Women’s University, Nara, 630-8506, Japan*

^5^*Graduate School of Organic Material Engineering, Yamagata University, Yonezawa, Yamagata, 992-8510, Japan*

^6^*Center for Integrative Medicine and Physics, Institute for Advanced Study, Kyoto University, Kyoto, 606-8501, Japan*

*^7^Physical Chemistry of Biosystems, Institute of Physical Chemistry, Heidelberg University, Heidelberg, D69120, Germany*

^8^*Department of Cancer Pathology, Faculty of Medicine, Hokkaido University, Sapporo, Hokkaido, 060-8638, Japan*

# Supplementary methods

Supplementary Scheme 1. Synthetic scheme of TEGPO ligand^1^. Reagents and conditions: (i) TsCl, pyridine, DCM; (ii) H_2_O_2_, DCM; (iii) BBr_3_, DCM; (iv) 1, K_2_CO_3_, DMF.

Synthesis of tetraethyleneglycol monomethyl ether tosylate (1). Tetraethyleneglycol monomethyl ether (4.17 g, 0.02 mol) was dissolved in super-dehydrated dichloromethane (100 ml). After cooling the solution to 0°C, pyridine (10 ml) was added under an argon atmosphere. After 15-minute stirring, *p*-toluenesulfonyl chloride (5.83 g, 0.03 mmol) was added under an argon atmosphere. The reaction solution was stirred overnight at 0°C and then quenched by 2% HCL aqueous solution. The mixture solution was stirred at room temperature for 1 hour. The product was extracted with dichloromethane and the organic layer was dried over anhydrous MgSO_4_ and filtered. The filtrate was concentrated under reduced pressure under 20°C, and the crude product was purified by silica gel column chromatography (silica gel, ethyl acetate) to give a colorless oil as the desired product (4.78 g, yield 66%).

^1^H-NMR (400 MHz, *d*_6_-DMSO): d/ppm = 7.80 (2H, d, *J* = 8.1 Hz, *m*-C*H*), 7.34 (2H, d, *J* = 8.1 Hz, *o*-C*H*), 4.16 (2H, t, *J* = 4.9 Hz, SOC*H*_2_), 3.72-3.51 (14H, m, C*H*_2_), 3.38 (3H, s, OC*H*_3_), 2.45 (3H, s, *p*-C*H*_3_).

Synthesis of tris(4-methoxyphenyl)phosphine oxide (2). Tris(4-methoxyphenyl)phosphine (5.03 g, 14.3 mmol) was dissolved in dichloromethane (100 ml), and 30% H_2_O_2_ aqueous solution (20 ml) was added to the solution. The mixture solution was stirred at 0°C for about 1 hour. Distilled water (100 ml) was then added and extracted with dichloromethane (100 ml) for two times. The organic layer was dried over anhydrous Na_2_SO_4_ and filtered. The filtrate was concentrated under reduced pressure to give white solid as the desired product (4.78 g, yield 91%).

^1^H NMR (400 MHz, CDCl_3_): d/ppm = 7.59–7.54 (6H, dd, *J* = 11.6, 8.7 Hz, Ar-*H*), 6.97–6.93 (6H, dd, *J* = 8.7, 2.3 Hz, Ar-*H*), 3.84 (3H, s, C*H*_3_).

^31^P NMR (162 MHz, CDCl_3_): d/ppm = 28.5 (s).

Synthesis of tris(4-hydroxyphenyl)phosphine oxide **(3).** Tris(4-methoxyphenyl)phosphine oxide (6.98 g, 19.0 mmol) was added to super-dehydrated dichloromethane (120 ml) under an argon atmosphere. 1M BBr_3_ dichloromethane solution (100 ml, 100 mmol) was added in 10 minutes under an argon atmosphere at -78°C. The mixture solution was returned to room temperature and stirred for 20 hours. Distilled water (200 ml) was added to quenching the reaction solution, and the precipitate was filtered. The precipitate was washed with sat. NaHCO_3_ aqueous solution and distilled water. The obtained solid was concentrated under reduced pressure to give white powder as the desired product (6.11 g, 18.7 mmol, yield 99%).

FT-IR (ATR): 3339 (br, O-H), 1116 (st, P=O) cm^-1^.

^1^H NMR (400 MHz, d_6_-DMSO): d/ppm = 10.1 (3H, s, O*H*), 7.36–7.31 (6H, dd, *J* = 11.2, 8.7 Hz, Ar-*H*), 6.87–6.84 (6H, dd, *J* = 8.7, 1.8 Hz, Ar-*H*).

^31^P-NMR (162 MHz, d_6_-DMSO): d/ppm = 26.1 (s).

ESI-Mass (*m/z*): [M+Na]^+^ calcd. for C_18_H_15_NaO_4_P, 349.06; found, 349.06.

Synthesis of **tris(4-((2,5,8,11-tetraoxatridecan-13-yl)oxy)phenyl)phosphine oxide** (TEGPO). Tris(4-hydroxyphenyl)phosphine oxide (0.28 g, 0.87 mmol) and K_2_CO_3_ (0.72 g, 5.23 mmol) were added to super-dehydrated DMF (30 ml) under an argon atmosphere. Tetraethyleneglycol monomethyl ether tosylate (1.01 g, 2.79 mmol) in super-dehydrated DMF (15 ml) were added to the solution under an argon atmosphere. Additional super-dehydrated DMF (20 ml) was added to improve the solubility. The reaction solution was stirred at 70°C for 3 days. After returning to room temperature, dichloromethane (70 ml) and 2% HCL aqueous solution (45 ml) was added. The product was extracted with dichloromethane and the organic layer was dried over anhydrous MgSO_4_ and filtered. The filtrate was concentrated under reduced pressure, and the crude product was purified by silica gel column chromatography (silica gel, ethyl acetate/methanol, v/v, 4:1) to give a light-yellow oil as the desired product (0.77 g, 0.86 mmol, yield 98%).

FT-IR (ATR): 1252 (st, C-O), 1112 (st, P=O), 1056 (st, C-O) cm^−1^.

^1^H-NMR (400 MHz, CDCl_3_): d/ppm = 7.57-7.49 (6H, dd, *J* = 11.4, 8.7 Hz, *o*-C*H*), 6.99-6.93 (6H, dd, *J* = 8.7, 2.3 Hz, *m*-C*H*), 4.18-4.14 (6H, t, *J* =4.8 Hz, PhOC*H*_2_), 3.89-3.84 (6H, t, *J* = 4.8 Hz, C*H*_2_), 3.75-3.70 (6H, m, C*H*_2_), 3.70-3.62 (24H, m, CH_2_), 3.56-3.52 (6H, m, C*H*_2_), 3.37 (9H, s, C*H*_3_).

^13^C-NMR (101 MHz, CDCl_3_): d/ppm = 161.5 (d, *J* = 2.9 Hz, *C_q_*O), 133.8 (d, *J* = 11.6 Hz, PC*C*), 124.5 (d, *J* = 110.8 Hz, P*C*), 114.5 (d, 13.5 Hz, *C*C_q_O), 71,9 (*C*H_2_), 70.8 (*C*H_2_), 70.6 (3×*C*H_2_), 70.4 (*C*H_2_), 69.5 (*C*H_2_), 67.4 (*C*H_2_), 59.0 (*C*H_3_).

^31^P-NMR (162 MHz, CDCl_3_): d/ppm = 28.7 (s).

Synthesis of [Eu(ntfa)_3_(H_2_O)_2_**].** [Eu(ntfa)_3_(H_2_O)_2_] was synthesized according to the published method^2^. 4,4,4-trifluoro-1-(2-naphthyl)-1,3-butanedione (4.88 g, 18.3 mmol) was dissolved in methanol (10 ml). Europium(III) acetate *n*-hydrate (2.13 g) in distilled water (50 ml) was dropwise added to the solution. An ammonia solution was then dropwise added to the mixture solution to reach pH 7. After stirring for 4 h at room temperature, the reaction mixture was dropwise added to distilled water to generate yellow precipitate. The mixture solution was stirred for 1 day to continue the precipitate generation. The precipitates were filtered, washed with hexane, and distilled water several times, dried in vacuo to obtain a yellow powder (4.30 g, 4.38 mmol, yield 72%).

FT-IR (ATR): 3059 (br, O-H), 1609 (st, C=O), 1289 (st, C-F) cm^−1^.

^19^F-NMR (376 MHz, CDCl_3_): d/ppm = −77.8 (s).

# Supplementary Results

**1. Interfacial properties of [Eu(ntfa)_3_(TEGPO)_2_] aggregate in** **water/methanol mixture**

The critical micelle-like aggregate concentration (CAC) of [Eu(ntfa)_3_(TEGPO)_2_] was measured at room temperature under systematic dilution of water/methanol mixture (90/10 v/v) in water. Sample solutions of 1 mM [Eu(ntfa)_3_(TEGPO)_2_] in water/methanol mixture (90/10 v/v) were initially prepared, and the surface tension was measured under systematic dilution of the sample solution in water using a KSV NIMA Langmuir-Blodgett trough (Biolin Scientific, Gothenburg, Sweden). As the reference, the surface tension of water/methanol mixture without [Eu(ntfa)_3_(TEGPO)_2_] was measured under systeatic dilution in water (Supplementary Figure 1, blue).

The surface excess (*Γ*) and the area per molecule at the interface (*A*) of [Eu(ntfa)_3_(TEGPO)_2_] were calculated from the slope (*B*) of the region that is free from the influence of the surface activity of methanol (*c* < 0.14 mM).

$$\Gamma=-\frac{B}{RT\ln10}=615 \left( {mmol}/{m^{2}} \right)$$

$$A=\frac{1}{\Gamma N_{A}}=270 \left( Å^{2} \right)$$

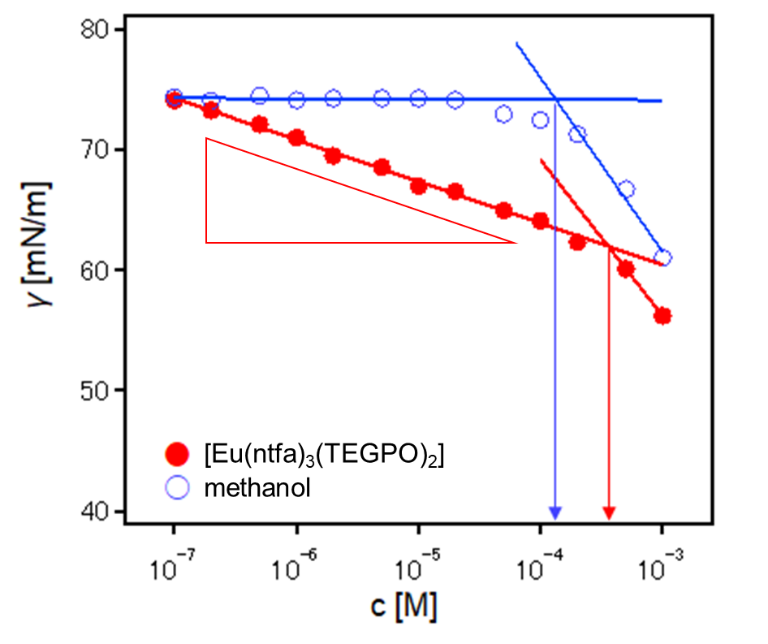


Supplementary Fig. 1 Surface tension measured under a series dilution of water/methanol mixture (90/10 v/v) in water, plotted as a function of [Eu(ntfa)_3_(TEGPO)_2_].

**2. Ultra-small and small angle X-ray scattering (USAXS/SAXS) profiles of [Eu(ntfa)_3_(TEGPO)_2_]**

The aggregation of micelle-like structure of [Eu(ntfa)_3_(TEGPO)_2_] at room temperature was measured to confirm the formation of aggregates in water/methanol mixture (90/10 v/v). Sample solutions of 1 mM [Eu(ntfa)_3_(TEGPO)_2_] in water/methanol mixture were initially prepared. From the USAXS/SAXS measurements, the aggregation size was estimated from the Guinier plot. The large aggregation radius, *R*_gL_, was approximately 150 nm and the small one, *R*_gs_, was approximately 0.5 nm. The small aggregation was due to TEGPO units. *A*_L_ and *A*_s_ was just constant.

$$I\left( q \right)=\frac{A_{L}}{3}R_{gL}^{2}q^{2}+\frac{A_{s}}{3}R_{gs}^{2}q^{2}$$

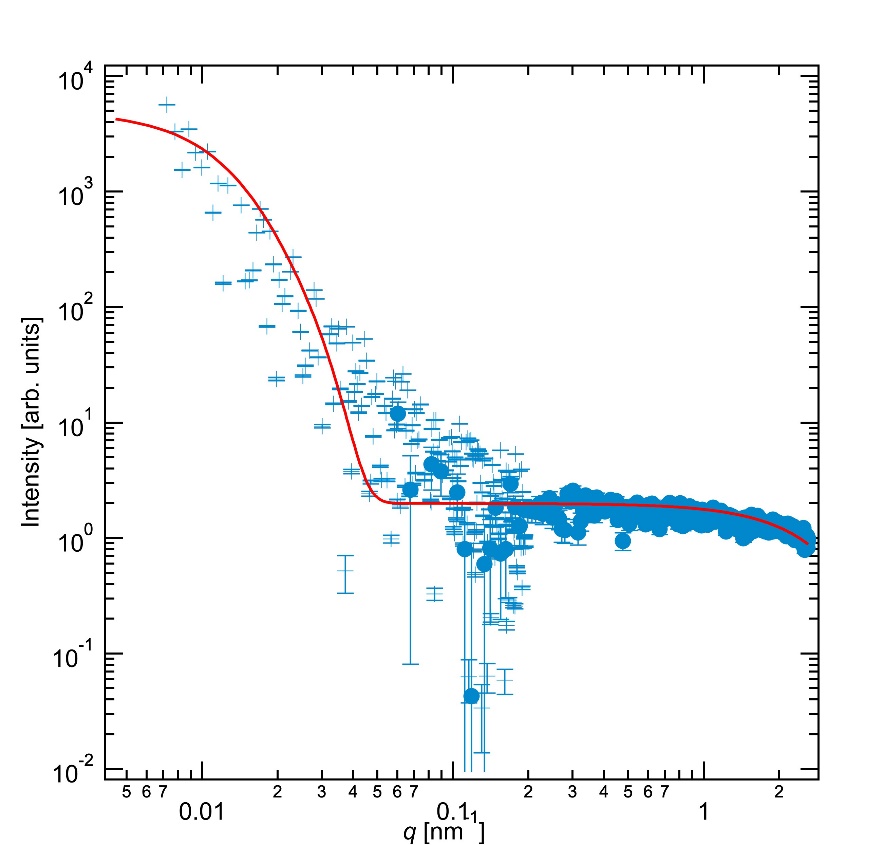


Supplementary Fig. 2 USAXS/SAXS profiles of [Eu(ntfa)_3_(TEGPO)_2_].

**3. DLS measurements of Triton X-100 micelles**

To compare with the aggregate size of [Eu(ntfa)_3_(TEGPO)_2_] in water/methanol mixture (90/10 v/v), Triton X-100 micelle with/without Eu(III) complexes were prepared. The concentrations were fixed to be 3 mM for Triton X-100 and 0.01 mM for Eu(III) complexes. According to DLS data, the micelle size is about 7.3 nm, independent of the encapsulated Eu(III) complexes. The aggregate size (370 nm) of [Eu(ntfa)_3_(TEGPO)_2_] in water/methanol mixture is 50 times larger than the micelle size.

(a) (b) (c)

Supplementary Fig. 3 Particle size distributions of (a) Triton X-100 micelle, (b) Triton X-100 micelle which encapsulates [Eu(ntfa)_3_(TEGPO)_2_], and (c) Triton X-100 micelle which encapsulates [Eu(ntfa)_3_(TPPO)_2_] in water/methanol mixture (90/10 v/v).

**4. Confocal microscope images**

NHA/TS

NHA/TSR

NHA/TSRA

3 h

6 h

8 h


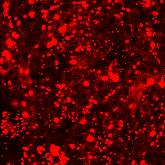

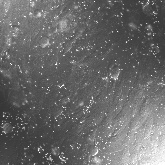

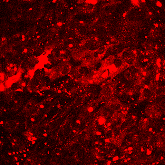

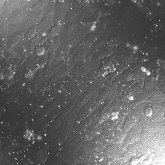

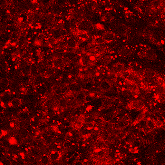

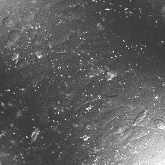

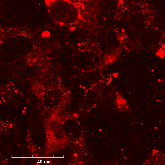

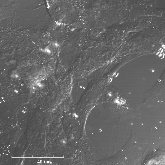

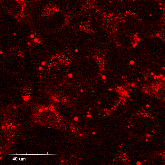

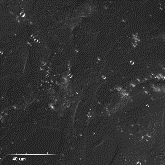

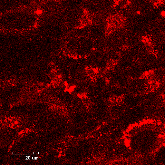

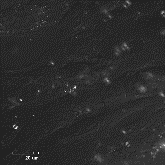

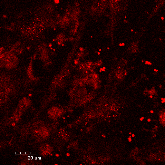

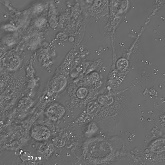

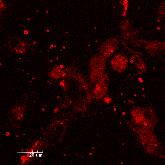

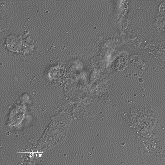

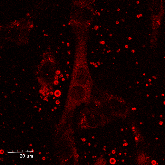

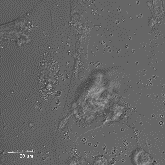


Supplementary Fig. 4 Confocal images of NHA cells treated with [Eu(ntfa)_3_(TEGPO)_2_] using confocal laser scanning microscope. Cells were incubated with 0.1 mM [Eu(ntfa)_3_(TEGPO)_2_], and luminescence and DIC images were obtained at 3, 6 and 8 hours.

**5. Uptake mechanism into cells**

To investigate the uptake mechanism of Eu(III) complex into cells, we investigated the uptake behavior of [Eu(ntfa)_3_(H_2_O)_2_] using U138 human brain tumor cells (glioblastoma). Initially, Eu(III) complex was added into culture medium of U138 cells, after 4-hour incubation, the cells were fixed with 3% paraformaldehyde, and endosomal marker EEA1 was stained with anti-EEA1 antibody (BD Biosciences, Franklin Lakes, NJ, USA), followed by Alexa Fluor 488-labeled secondary antibody (Invitrogen). We observed vesicle-derived luminescence characteristic of endocytosis within 1 hour after addition of Eu(III) complex (Supplementary Fig. 5a). On the other hand, on the EEA1-staining images, the position of Eu(III) complex luminescence matched well with those of EEA1, indicating the uptake of Eu(III) complex *via* endosomes (Supplementary Fig. 5b). The pre-treatment with a dynamin inhibitor, known for its role as an endocytosis inhibitor, resulted in the abrogation of EEA1 staining and the corresponding uptake of Eu(III) complex (Supplementary Fig. 5c).

(a)
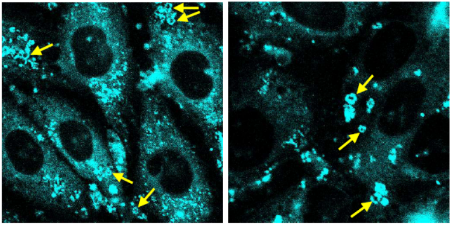


(b)
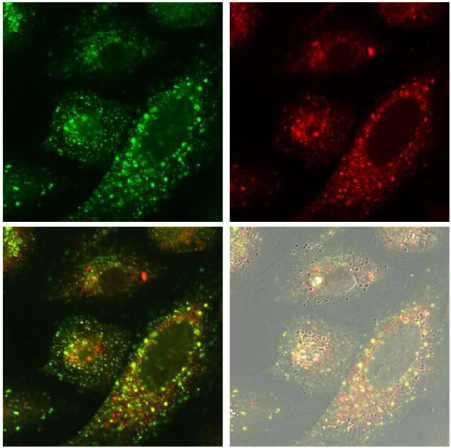
 (c)
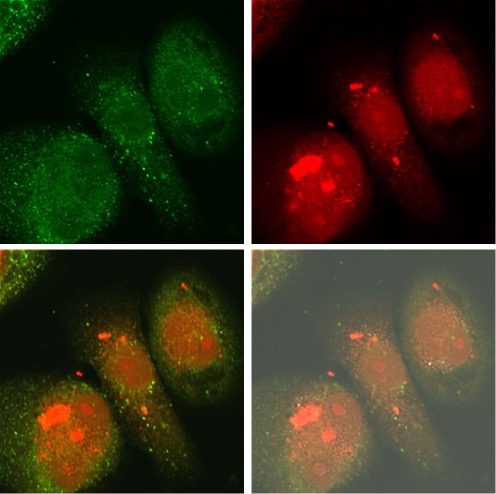


Supplementary Fig. 5 Uptake mechanism of Eu(III) complex into cells, along with luminescence images under different treatment conditions. Luminescence images of cells (a) treated with Eu(III) complex, (b) co-staining with EEA1 in green and Eu(III) complex in red without dynamin inhibitor, and (c) co-staining with EEA1 in green and Eu(III) complex in red with dynamin inhibitor.

**6. Cellular emission spectra and controlled experiments**

(a) (b) (c)

Supplementary Fig. 6 Emission spectra of [Eu(ntfa)_3_(TEGPO)_2_] in (a) NHA/TS (benign grade II gliomas), (b) NHA/TSR (malignant grade III gliomas) and (c) NHA/TSRA (malignant grade IV gliomas) cells. Excitation wavelength: 380 nm with slit of 1 nm bandpass. Detection side: 530 nm long-pass filter, slit of 1~3 nm bandpass.

Supplementary Fig. 7 The time course of *k*_r_ for [Eu(ntfa)_3_(TEGPO)_2_] in DMEM medium and the cell suspensions of tumor cells.

**7. The evaluation of the cytotoxic potential of [Eu(ntfa)_3_(TEGPO)_2_]**

To assess the cytotoxicity of [Eu(ntfa)_3_(TEGPO)_2_] in tumor cells, the cell proliferation assay under [Eu(ntfa)_3_(TEGPO)_2_] treatment was conducted using an alternative method known as Cell Counting Kit-8 (CCK-8, Dojindo, Japan). The CCK-8 assay has good correlation with the MTT assay. The CCK-8 is highly sensitive living cell count measurement kit that uses the water-soluble tetrazolium salt WST-8 as a coloring reagent. NADH produced by dehydrogenase in cells reduces WST-8 to orange formazan via 1-Methyoxy PMS, and the amount of the formazan dye is proportional to the number of viable cells.

In the CCK-8 assay, cell viabilities with Eu(III) treatment were estimated to be 59.0% for NHA/TS, 59.8% for NHA/TSR, 54.1% for NHA/TSRA after T = 3 hours, 53.8% for NHA/TS, 51.9% for NHA/TSR, 50.7% for NHA/TSRA after T = 6 hours and 43.0% for NHA/TS, 40.6% for NHA/TSR, 37.1% for NHA/TSRA after T = 9 hours. The percentage of viable cells was in the order TS>TSR>TSRA, which correlates with the Eu(III) complex uptake data. The CCK-8 assay analysis suggested (i) **[Eu(ntfa)_3_(TEGPO)_2_]** might exhibit slight toxicity when a substantial amount of the complex is uptaken, and (ii) the occurrence of cell death with long-time (after T = 3 hours) Eu(III) complex treatment.

Supplementary Fig. 8 Cell counting Kit-8 assay.

**8. Flow cytometry**

Flow cytometry was performed to analyze cell cycle distribution and the percentage of dead cells after **[Eu(ntfa)_3_(TEGPO)_2_]** treatment. In Supplementary Fig. 9, it suggested the Eu(III) complex affected cell cycle distribution, leading to decreases in G2/M phase (green color) for both NHA/TS and NHA/TSRA, while an increase for NHA/TSR. The increase in NHA/TSR cell cycle distribution at G2/M phase may be due to abnormalities in cytokinesis and then be on track to die. On the other hand, at T = 3 → T = 6 → T = 9 hours, the number of dead cells (apoptotic cells) were evaluated as 4.1% → 2.7% → 3.1% for NHA/TS, 0.9% → 5.4% → 3.2% for NHA/TSR and 1.2% → 2.6% → 8.8% for NHA/TSRA. Thus, Eu(III) complex affected tumor cell cycle distribution and lead to increases in cell death.


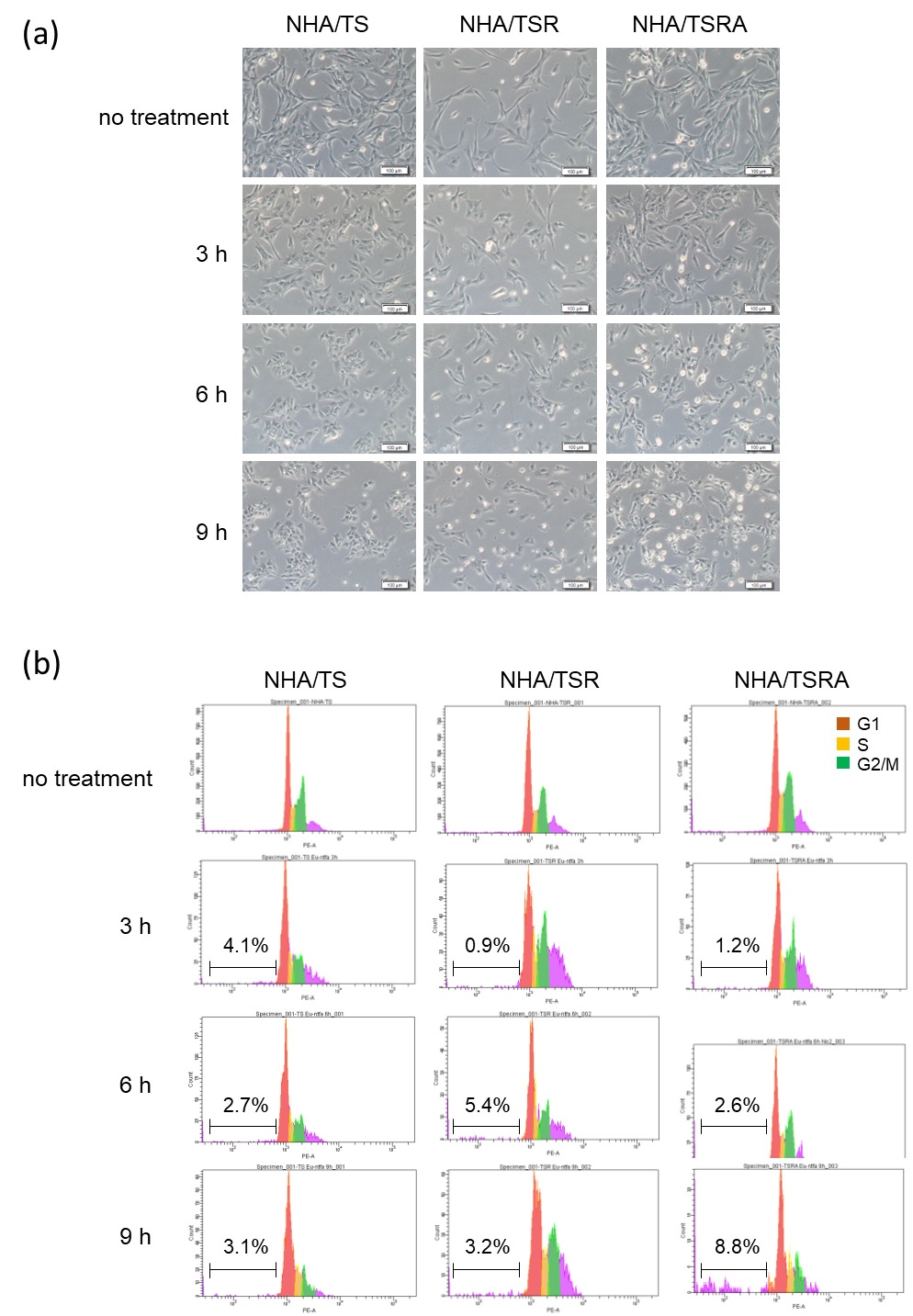


Supplementary Fig. 9 (a) Microscopy images of NHA cells treated w/ and w/o [Eu(ntfa)_3_(TEGPO)_2_]. (b) Flow cytometry measurements.

# Supplementary References

1. Kemper, B. et al. Reversible covalent and supramolecular functionalization of water-soluble gold(I) complexes. *Chem. Eur. J.* **23**, 6048-6055 (2017).

2. Koizuka, T. et al. Photosensitized luminescence of highly thermostable mononuclear Eu(III) complexes with π-expanded β-diketonate ligands. *Bull. Chem. Soc. Jpn.* **90**, 1287-1292 (2017).
